# Role of the Aryl Hydrocarbon Receptor and Gut Microbiota-Derived Metabolites Indole-3-Acetic Acid in Sulforaphane Alleviates Hepatic Steatosis in Mice

**DOI:** 10.3389/fnut.2021.756565

**Published:** 2021-10-13

**Authors:** Xiuxiu Xu, Siyuan Sun, Ling Liang, Chenxi Lou, Qijin He, Maojuan Ran, Lu Zhang, Jingyue Zhang, Chen Yan, Hengjie Yuan, Lu Zhou, Xin Chen, Xin Dai, Bangmao Wang, Jie Zhang, Jingwen Zhao

**Affiliations:** ^1^Tianjin Key Laboratory of Digestive Diseases, Department of Gastroenterology and Hepatology, Tianjin Institute of Digestive Disease, Tianjin Medical University General Hospital, Tianjin, China; ^2^NHC Key Laboratory of Hormones and Development, Tianjin Key Laboratory of Metabolic Diseases, Tianjin Medical University Chu Hsien-I Memorial Hospital & Tianjin Institute of Endocrinology, Tianjin Medical University, Tianjin, China; ^3^Department of Pharmacy, Tianjin Medical University General Hospital, Tianjin, China

**Keywords:** NAFLD, AHR, indole-3-acetic acid, sulforaphane, gut microbiota, high-fat diet

## Abstract

**Scope:** Gut microbiome-derived metabolites are the major mediators of diet-induced host-microbial interactions. Aryl hydrocarbon receptor (AHR) plays a crucial role in glucose, lipid, and cholesterol metabolism in the liver. In this study, we aimed to investigate the role of indole-3-acetic acid (IAA) and AHR in sulforaphane (SFN) alleviates hepatic steatosis in mice fed on a high-fat diet (HFD).

**Methods and Results:** The HFD-fed male C57BL/6 mice were intervened with SFN for 6 weeks. HFD-mice showed classical pathophysiological characteristics of hepatic steatosis. The results showed that SFN significantly reduced body weight, liver inflammation and hepatic steatosis in HFD-fed mice. SFN reduced hepatic lipogenesis by activating AHR/SREBP-1C pathway, which was confirmed in HepG2 cell experiments. Moreover, SFN increased hepatic antioxidant activity by modulating Nrf-2/NQO1 expression. SFN increased serum and liver IAA level in HFD mice. Notably, SFN manipulated the gut microbiota, resulting in reducing *Deferribacteres* and proportions of the phylum *Firmicutes/Bacteroidetes* and increasing the abundance of specific bacteria that produce IAA. Furthermore, SFN upregulated *Ahr* expression and decreased the expression of inflammatory cytokines in Raw264.7 cells.

**Conclusions:** SFN ameliorated hepatic steatosis not only by modulating lipid metabolism via AHR/SREBP-1C pathway but regulating IAA and gut microbiota in HFD-induced NAFLD mice.

## Introduction

Approximately 25% of the global population struggles with non-alcoholic fatty liver disease (NAFLD) with a dramatically growing prevalence (1). Currently, the widely accepted pathogenesis of NAFLD is that lipid deposits accumulate in the liver, followed by the activation of immune cells and the production of proinflammatory cytokines (2, 3). Regulating metabolic nuclear receptors, such as the sterol regulatory element-binding protein-1c (Srebp-1c), carbohydrate response element-binding protein (ChREBP), and peroxisome proliferator-activated receptor γ (PPAR-γ) prevents hepatic steatosis caused by a high-fat diet (HFD), indicating that uncontrolled *de novo* lipogenesis contributes to the development of NAFLD (4–6).

Increasing numbers of studies have demonstrated that Western and high-calorie diet influences the composition of the gut microbiota (7), which in turn has gained attention with respect to metabolic diseases, such as obesity (8), metabolic syndrome (MS) (9), diabetes (10), cardiovascular disease (11), and NAFLD (12). A myriad of studies has reported the effects of gut microbial metabolites on host health and disease, which might be mediated partially through the metabolome, such as short-chain fatty acid, serotonin, bile acids, and tryptophan metabolites (13). Especially the indole-3-acetic acid (IAA) that is mainly synthesized by *Clostridium, Bacteroides*, and *Bifidobacterium* (14), participates in the remission of NAFLD by improving insulin resistance, oxidative stress, and lipid metabolism (3, 15). Indole and its derivatives maintain the integrity of the liver and benefit intestinal permeability and immune function, which might inhibit liver inflammation (16). Additionally, TH17/regulatory T (Treg) cells balance plays a major role in autoimmune and inflammatory diseases. Notably, previous studies reported that tryptophan catabolite promotes the differentiation of naive CD4^+^ T helper cells into Treg cells and TH17 cells via aryl hydrocarbon receptor (AHR) (17).

AHR is a ligand-activated transcription factor widely expressed in all types of tissues and cells, especially immune cells (18). Furthermore, AHR participates in several physiological processes, including chemical and microbial defense, cell proliferation, immunity, inflammation, and energy metabolism *via* ligand binding and regulating target genes (19). Gut microbiome-derived metabolites, such as endogenous ligands of AHR alleviate gastrointestinal inflammation, atopic dermatitis, and arthritis (20–23). Accumulating evidence suggested that AHR plays a crucial role in glucose, lipid, and cholesterol metabolism in the liver (24), thereby deeming its role in the pathogenesis of NAFLD. Moreover, Kupffer cells are resident liver macrophages and the primary cells that produce various inflammatory and fibrosis mediators, play a major role in liver inflammation (25). Previous studies have also shown that the AHR agonist IAA reduces the level of proinflammatory cytokines produced by macrophages stimulated by fatty acids and LPS and inhibits the migration of macrophages to chemokines, thereby indicating that AHR and ligands regulate the macrophages (3).

Phytochemicals widely present in a variety of fruits and vegetables can prevent lipid-related diseases (26). Sulforaphane (SFN), a natural isothiocyanate compound extracted from cruciferous vegetables, exerts strong anti-inflammatory and anti-cancer effects (27). It also alleviates alcohol-induced hepatosteatosis and HFD-induced lipid accumulation in mice (28). However, whether SFN improves gut microbiota and its metabolites is yet to be elucidated.

Therefore, the objective of the present study is to test the hypothesis that SFN might regulate IAA and gut microbiota in HFD-fed mice and prevent NAFLD through activating AHR/SREBP-1C pathway.

## Materials and Methods

### Animals and Treatment

Male C57BL/6 mice (3–4weeks) were housed in a specific-pathogen-free (SPF) environment at the Laboratory Animal Center of Chinese Academy of Medical Sciences Institute of Radiation Medicine, with a 12 h light/dark cycle and access to food and water freely. The animal chow was purchased from Hua Fu Kang (Beijing, China). After acclimation for 1 week, the mice were randomly classified into two weight-matched groups: (1) normal chow diet (NCD) group (*n* = 10/group); (2) high-fat diet (HFD) group (*n* = 20/group) (HFD: protein 18.1%, fat 61.6%, carbohydrates 20.3%). After 22 weeks of the initial feeding period, HFD group mice were randomly assigned to two groups (*n* = 10/group): a HFD with saline, and a HFD with Sulforaphane (purity >99%, *Item NO.10496*, Cayman) 25 mg/kg intervention by gavage daily for 6 weeks. The liver tissues were harvested, weighed, and stored at −80°C for further analysis. All animal welfare and experimental procedures complied with the Laboratory Animal Management Regulations in China.

### Biochemical Analysis

Following anesthetization, blood was collected from the retro-orbital plexus, and the serum was separated and stored at −80°C. The levels of alanine aminotransferase (ALT), aspartate aminotransferase (AST), cholesterol (TC), and triglyceride (TG) in serum were measured by standard procedures in the Institute of Clinical Chemistry of the Tianjin Medical University General Hospital.

### Histopathology

The liver tissue specimens were immediately fixed in 4% formaldehyde, embedded in paraffin, sectioned into 4-μm-thick slices, and stained with hematoxylin-eosin (H&E) for histological examination of fat droplets. The stained tissues were assessed by a pathologist in a blinded manner, and images were captured using Leica fluorescence microscope (Leica, Germany).

### Cell Culture and Treatment

Raw264.7 and HepG2 cells were obtained from the Chinese Academy of Sciences Cell Bank (Shanghai, China). Raw264.7 murine macrophages were cultured in Dulbecco's modified Eagle's medium (DMEM, Gibco) supplemented with 10% fetal bovine serum (FBS, Gibco) and 1% nonessential amino acid solution (Solarbio, Beijing, China). Then, the cells were pretreated with Sulforaphane (SFN, purity >99%) 20 μM for 6 h, followed by 300 μM palmitate (PA, Sigma Aldrich) complexed with Bovine serum albumin (BSA, #abs9156, Absin Bioscience Inc.) treatment. After 18 h, 10 ng/mL lipopolysaccharide (LPS, from Escherichia coli, O111:B4, Sigma Aldrich) was added to the culture medium, and the cells were incubated for an additional 6 h. HepG2 cells were cultured in DMEM supplemented with 10% FBS. After reaching 70% confluency, cells were stimulated with 10 μM SFN or 500 μM indole-3-acetic acid (IAA, purity: 99.1%, Sigma Aldrich) for 24 h, followed by treatment with or without 250 μM PA for another 24 h in serum-free media containing 25 mM glucose and 0.25% BSA. All cells were incubated at 37°C in a humidified atmosphere containing 5% CO_2_.

### RNA Isolation and RT-qPCR Analysis

Total RNA was purified from liver or cells using TRIzol (Invitrogen, Carlsbad, CA, USA.) and reverse transcribed to cDNA using TIANScript RT kit (Tiangen Inc., Beijing, China). Subsequently, quantitative PCR amplification was performed using SYBR^TM^ Select Master Mix on the ABI StepOne Plus Real-Time PCR System (Applied Biosystems). Mir-155 was detected by Bulge-Loop^TM^ miRNA qRT-PCR Starter Kit (Ribobio, Guangzhou, China). The relative mRNA or miRNA expression levels were calculated by normalizing the level of the target genes against that of the housekeeping genes, such as glyceraldehyde-3-phosphate dehydrogenase (*GAPDH*), using the 2^−ΔΔCt^ method. The specific primer sequences are listed in Table 1.

**Table 1:** Primer sequences used in RT-qPCR.

**Gene**	**Forward sequence (5^′^ → 3^′^)**	**Reverse sequence (5^′^ → 3^′^)**
*Gapdh*	GGAGAAACCTGCCAAGTATG	TGGGAGTTGCTGTTGAAGTC
*Mcp1*	TTAAAAACCTGGATCGGAACCAA	GCATTAGCTTCAGATTTACGGGT
*Il-1β*	GTGTCTTTCCCGTGGACCTT	AATGGGAACGTCACACACCA
*Il-6*	CCAGTTGCCTTCTTGGGACT	GGTCTGTTGGGAGTGGTATCC
*F4/80*	CTTTGGCTATGGGCTTCCAGTC	GCAAGGAGGACAGAGTTTATCGTG
*Tnf-α*	GGTGCCATGTCTCAGCCTCTT	GCCATAGAACTGATGAGAGGGAG
*Srebp-1c*	GGAGCCATGGATTGCACATT	GGCCCGGGAAGTCACTGT
*Acc1*	GACAGACTGATCGCAGAGAAAG	TGGAGAGCCCCACACACA
*Scd1*	TTCTTGCGATACACTCTGGTGC	CGGGATTGAATGTTCTTGTCGT
*Fas*	GGAGGTGGTGATAGCCGGTAT	TGGGTAATCCATAGAGCCCAG
*Gclm*	TGACTCACAATGACCCGAAA	CTTCACGATGACCGAGTACCT
*Nqo1*	AGCGTTCGGTATTACGATCC	AGTACAATCAGGGCTCTTCTCG
*Gclc*	AGATGATAGAACACGGGAGGAG	TGATCCTAA AGCGATTGTTCTTC
*Gstm1*	GCAGCTCATCATGCTCTGTT	CATTTTCTCAGGGATGGTCTTC
*GAPDH*	CCCTTCATTGACCTCAACTACATGG	CATGGTGGTGAAGACGCCAG
*SREBP-1C*	CTTCCGCCCTTGAGCTG	CTGGTGTGTCCGTGTGG
*NQO1*	TGGTTTGGAGTCCCTGCCAT	CACTGCCTTCTTACTCCGGAAGG
*ACC1*	ATGTCTGGCTTGCACCTAGTA	CCCCAAAGCGAGTAACAAATTCT
*FAS*	CCGCGGTTTAAATAGCGTCG	CACCTCCTCCATGGCTGGT

### Protein Extraction and Western Blot Analysis

Total protein was extracted by RIPA buffer (Solarbio, Beijing, China) containing protease inhibitors (PMSF) (Solarbio, Beijing, China), while nuclear protein was extracted using a Nuclear Protein Extraction kit (Solarbio, Beijing, China). The protein concentrations were determined using a BCA Protein Assay kit (Solarbio, Beijing, China). The tissue lysates were separated by sodium dodecyl sulfate-polyacrylamide gel electrophoresis (SDS-PAGE) and transferred to nitrocellulose membranes (Pall Co, USA) at a voltage of 90V. Blotted membranes were blocked with 5% BSA for 1.5 h at room temperature and incubated overnight at 4°C with anti-FAS(#3180, Cell Signaling Technology), anti-SREBP-1(#NB100-2215, Novus), anti-NRF2(#12721, Cell Signaling Technology), anti-AHR (#sc-13308, Santa Cruz Biotechnology), anti-Lamin-B2(#12255, Cell Signaling Technology) and anti-β-Actin(#3700, Cell Signaling Technology), followed by incubation with horseradish peroxidase-conjugated goat anti-rabbit or anti-mouse IgG (1:5,000, Zhongshan Golden Bridge Biotechnology, Beijing, China) for 1 h at room temperature. Fluorescent bands were visualized and photographed using an SYNGENE CGQ/D2 GEL-Image System (Bio-Rad, America).

### Targeted Metabolomics

Briefly, 100 μL of mouse serum samples were mixed with 10 μL indole-3-acetic-2,2-d2 acid (D2-IAA, purity: 98%, Sigma Aldrich) 100 ng/mL, and 400μL chilled methanol. The mixture was agitated for 3 min, and the supernatant was collected by centrifugation at 14,000 rpm for 10 min. The liver tissues (100 mg) were homogenized with a grinding rod and mixed with 10 μL D2-IAA (100 ng/mL) and 500 μL chilled methanol. The mixture was vortexed for 3 min, and the supernatant was collected by centrifugation at 6,800 rpm for 10 min at 4°C four times with an interval of 45 s each. Subsequently, the supernatant liquor/0.01% formic acid solution (1:9, v/v) was transferred to a new tube, vortexed for 1 min, and spun at 14,000 rpm for10 min at 4°C. Finally, 10 μL supernatant liquor was analyzed by ultra-high-performance liquid chromatography-tandem mass spectrometry (UPLC-MS/MS) system. The metabolite levels were normalized to the sample weight.

### Immunofluorescence and Immunohistochemistry

Liver tissues were cut, deparaffinized, dehydrated, and incubated with specific antibodies against F4/80(#70076, Cell Signaling Technology) overnight at 4°C. Subsequently, the slides were washed with phosphate-buffered saline (PBS) three times and incubated with fluorochrome-conjugated secondary antibody for 1 h at room temperature in the dark. Subsequently, the slides were washed and incubated with Prolong Gold antifade containing DAPI and mounted for IF. IHC was carried out to determine the expression of F4/80 in the liver tissue. The slides were analyzed under a Leica fluorescence microscope (Leica, Germany).

### *16S* rRNA Gene Sequencing

The composition of mice feces was analyzed by*16S* rRNA gene amplicon sequencing. The extracted genomic DNA was analyzed by 1% agarose gel electrophoresis, and the product was purified using Agencourt A MPure XP Nucleic Acid Purification kit (Beckman Coulter). The V3 and V4 regions of the *16S* gene were amplified by PCR and sequenced on an Illumina MiSeqPE300. Quantitative insights into microbial ecology (QIIME) software pipeline v.1.8.0 was used to analyze the raw data files. Operational Taxonomic Units (OTUs) were used clustered into represent groups and assigned to taxonomy using VSEARCH 2.7.1 at 97% similarity. Then, a representative sequence of OUT was subjected to the taxonomy analysis basing on the Sliva bacterial 16S rRNA database. Alpha-diversity (Chao1, Observed_species, PD_whole_tree, Shannon) and beta-diversity were analyzed to identify the species diversity. The relative abundance of bacteria was expressed at the percentage.

### RNA *in situ* Hybridization

RNAscope Multiplex Fluorescent Reagent kit v2 (ACDbio) in combination with Opal 570 Reagent Pack (Perkin Elmer) were used for RNA *in situ* hybridization according to the manufacturer's instructions. Before mounting, the slides were counterstained with a primary antibody against F4/80 for 1 h at room temperature and processed for IF detection. Probes for murine AHR were used (ACDbio).

### Statistical Analysis

Data are represented as mean ± standard deviation (SD). GraphPad Prism 7 (GraphPad Software Inc., San Diego, CA, USA) was used for statistical analysis. Significant differences were analyzed using one-way ANOVA in multiple groups. *P* < 0.05 were considered as statistically significant.

## Results

### SFN Attenuated Hepatic Steatosis in HFD-Induced NAFLD Mice

**Figure 1:**
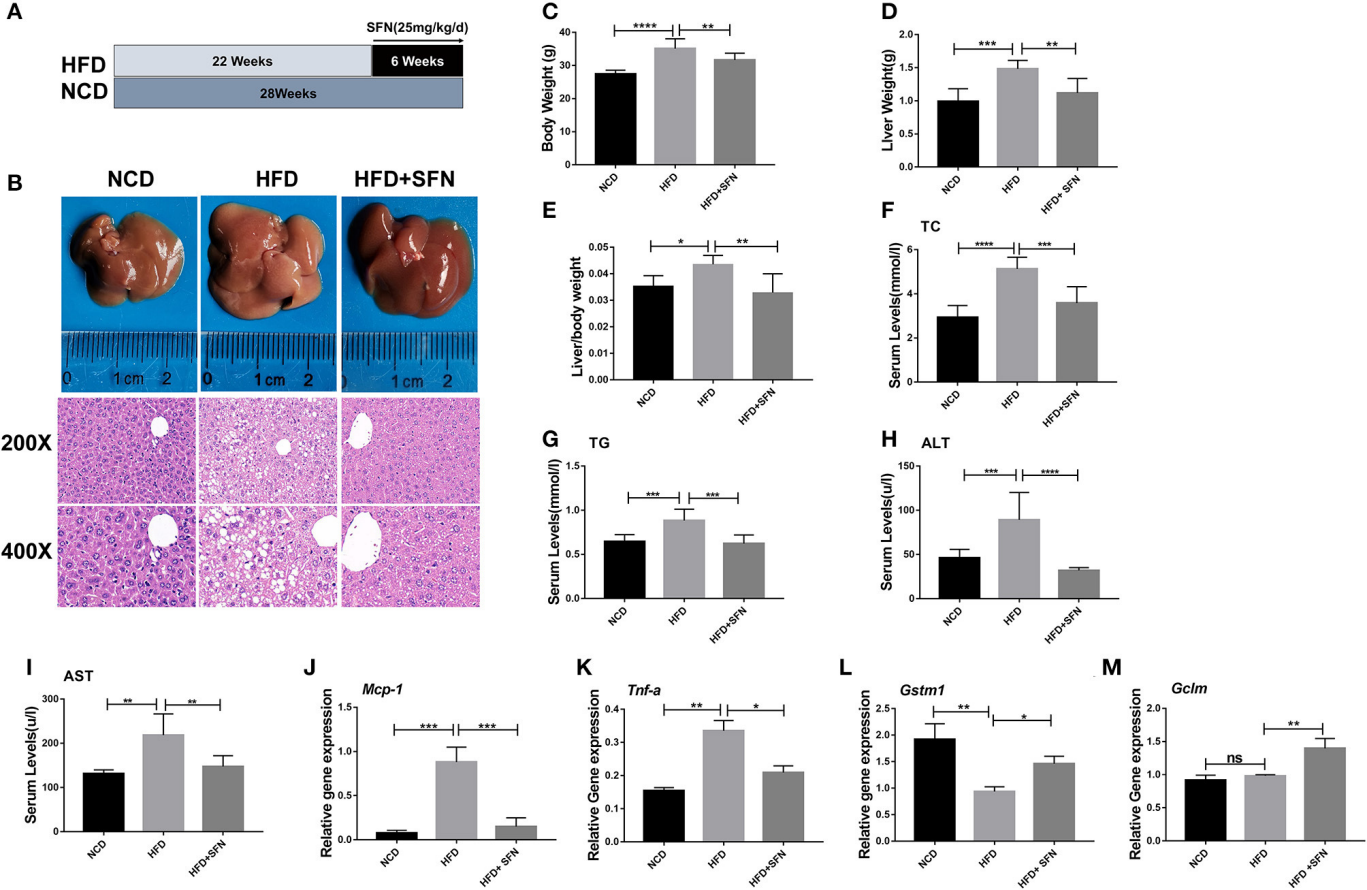
Preventive effects of SFN on the development of HFD-induced NAFLD in mice. **(A)** Model development and treatment. **(B)** Representative liver images and H&E staining of liver sections from different groups (×400). **(C–E)** The mice body weight, liver weight and index (liver/body weight). **(F–I)** Serum levels of TC, TG, ALT and AST. **(J,K)** The expression of *Mcp-1* and *Tnf-a* in the liver. **(L,M)** SFN attenuates the expression of anti-oxidative stress mRNA. Data are presented as mean ± SD. ns, not significant, *P < 0.05, **P < 0.01, ***P < 0.001, ****P < 0.0001 vs. HFD group.

To address whether SFN curbs the progression of the disease, we used HFD-fed mice as the NAFLD model (Figure 1A). Representative liver photographs of mice are shown in Figure 1B. The liver tissues of the HFD group showed severe hepatic steatosis with marked fat droplets and balloon-like structures in the hepatocytes by H&E staining, while the administration of SFN caused a remarkable amelioration of the appearance of hepatic steatosis (Figure 1B). In addition, body weight, liver weight and index (liver/body weight) of the mice were increased by HFD, which were also restored by SFN treatment (Figures 1C–E). Similarly, the level of TC and TG in serum were largely reduced by SFN. SFN also exhibited protective effects against the HFD-induced liver damage, which was further supported by low serum AST and ALT levels (Figures 1F–I). Furthermore, SFN decreased mRNA expression of tumor necrosis factor-alpha (*Tnf-a*) and monocyte chemoattractant protein-1 (*Mcp-1*) in liver tissues (Figures 1J,K), suggesting that it significantly alleviates liver inflammation. Notably, the expression of anti-oxidative stress-related genes *Gstm1* was inhibited by HFD, but *Gstm1* (*P* < 0.05) and *Gclm* (*P* < 0.01) were upregulated by SFN treatment (Figures 1L,M). Thus, these data revealed that SFN drastically alleviates HFD-induced NAFLD.

### SFN Administration Improved Lipid Metabolism of the Liver in HFD-Fed Mice

**Figure 2:**
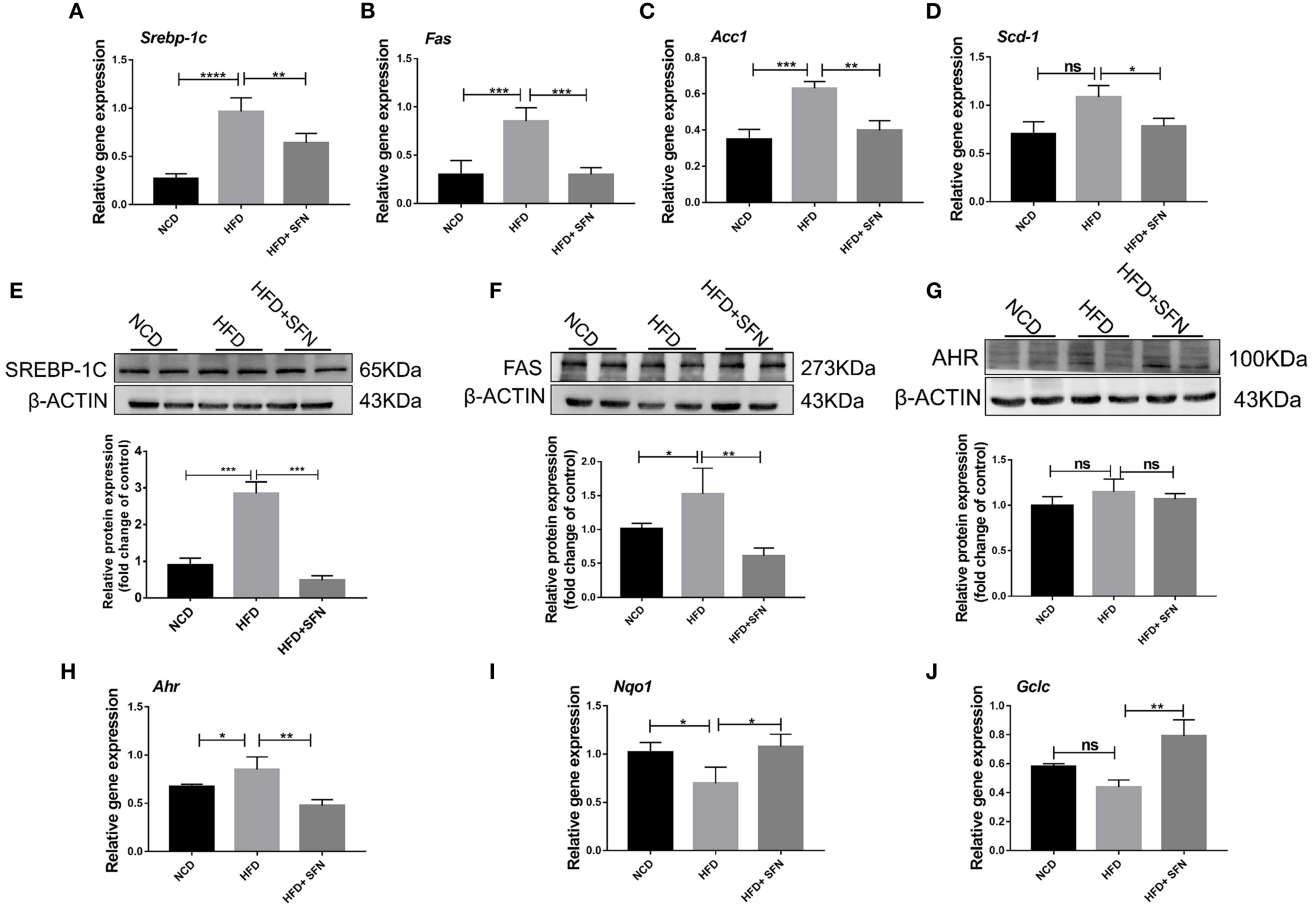
Effects of SFN on lipid metabolism of the liver. **(A–D)** Relative mRNA levels of *Srebp-1c, Fas, Acc1* and *Scd-1* were associated with *de novo* lipogenesis in the liver. **(E,F)** Western blot showing SREBP-1C and FAS protein levels in the liver. **(G)** Levels of total AHR protein in the liver. **(H)** The expression of *Ahr* in the liver tissue. **(I,J)** Antioxidant stress relative gene expression in the liver. Data are presented as mean ± SD. ns, not significant, *P < 0.05, **P < 0.01, ***P < 0.001, ****P < 0.0001 vs. HFD group.

Herein, we assessed whether SFN displays a protective effect on hepatic lipid metabolism and observed that the expression of fatty acid metabolism genes, sterol regulatory element-binding protein-1c (*Srebp-1c*), fatty acid synthase (*Fas*), stearoyl-CoA desaturase-1 (*Scd-1*), and acetyl-CoA carboxylase 1 (*Acc1*) in the liver, was decreased by SFN administration (Figures 2A–D). SREBP-1C is a transcription factor involved in hepatic lipid metabolism and regulation of the expression of multiple genes in TG and fatty acid synthesis (29). Furthermore, we examined the expression of SREBP-1C and FAS in the liver by Western blot and found that the levels of both proteins were reduced by SFN (Figures 2E,F). Moreover, SFN had no effect on endogenous levels of total AHR protein, but markedly reduced the mRNA expression of *Ahr* in the liver tissue of HFD mice (*P* < 0.01) (Figures 2G,H). We also observed that SFN significantly elevated the expression of *Nqo1* (*P* < 0.05) and *Gclc* (*P* < 0.01) (Figures 2I,J).

### SFN Alleviates Lipid Metabolism in Hepatocytes by Activating AHR

**Figure 3:**
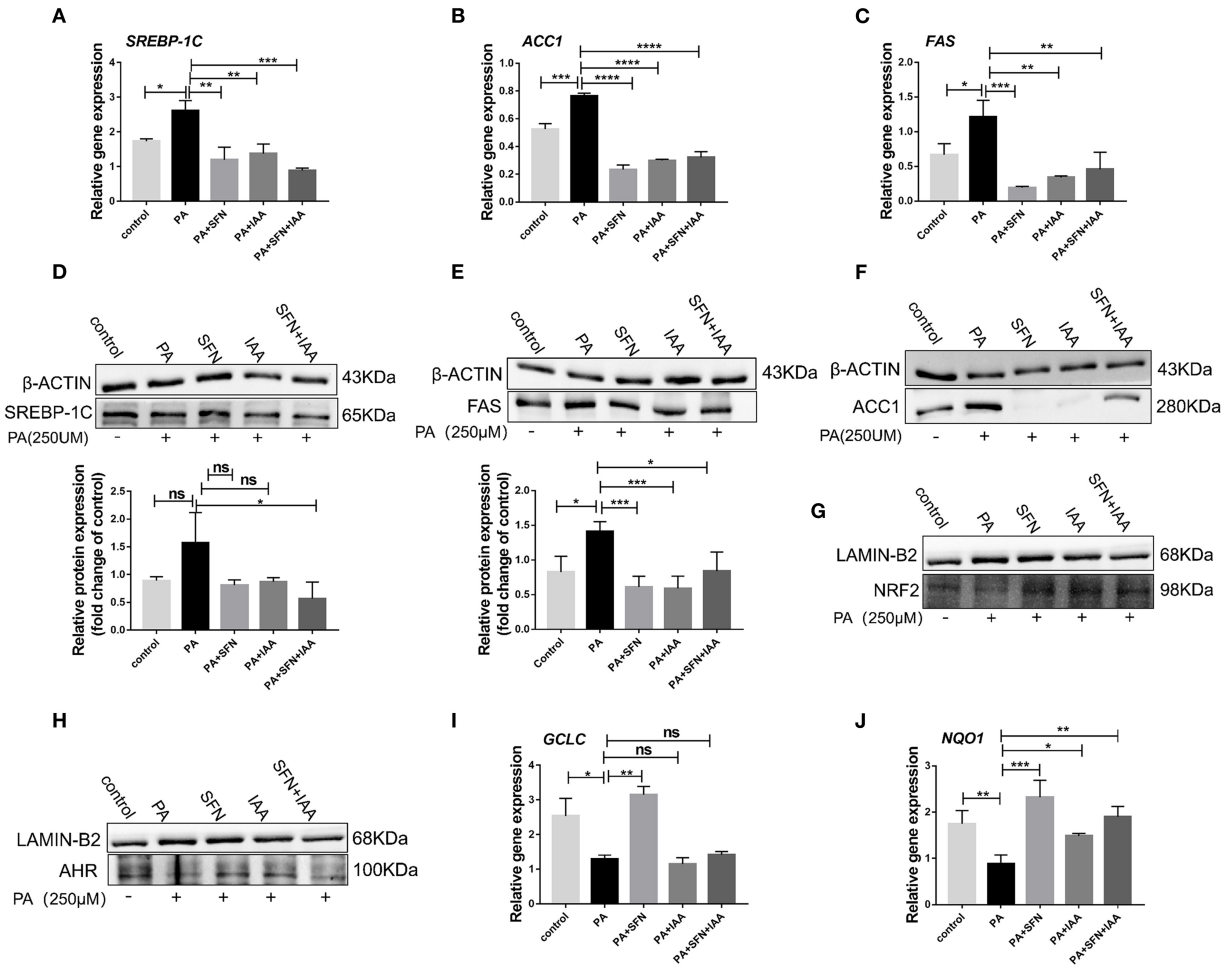
SFN attenuates the expression of genes related to the *de novo* lipogenesis in HepG2 cells. **(A–C)** SFN decreased the mRNA expression of *SREBP-1C, ACC1*, and *FAS* in HepG2 cells exposed to PA. **(D–F)** SFN decreased the protein expression of SREBP-1C, FAS, and ACC1 in HepG2 cells exposed to PA. **(G,H)** Immunoblots showing NRF2 and AHR content in the nuclear fractions. **(I,J)** Antioxidant stress relative gene expression in HepG2 cells. Quantitation of results of Western blot using Image J software. Data are presented as mean ± SD. ns means not significant, *P < 0.05, **P < 0.01, ***P < 0.001, ****P < 0.0001 vs. HFD group.

To further explore the effect of AHR on lipid metabolism, relevant experiments were conducted *in vitro* with HepG2 cells. Intriguingly, SFN also reduced the protein levels of SREBP-1C, ACC1, and FAS, and their mRNA expression in HepG2 cells (Figures 3A–F), which was consistent with the data in mice. Especially, the protein level of ACC1 was potently reduced by SFN. Furthermore, treatment with SFN increased the nuclear protein content of AHR and NRF2 compared to the PA-treated group (Figures 3G,H). We also observed that SFN significantly reduces the expression of NRF2 target genes: *NQO1* (*P* < 0.001) and *GCLC* (*P* < 0.01) (Figures 3I,J). In addition, IAA was used as a positive control, and the results of the SFN group were like those of the IAA-positive group compared to the PA-treated cells. Taken together, SFN inhibited the progression of NAFLD by activating the AHR/SREBP-1c pathway.

### Changes in Gut Microbiota Composition and Species Diversity During SFN-Treatment in HFD-Fed Mice

**Figure 4:**
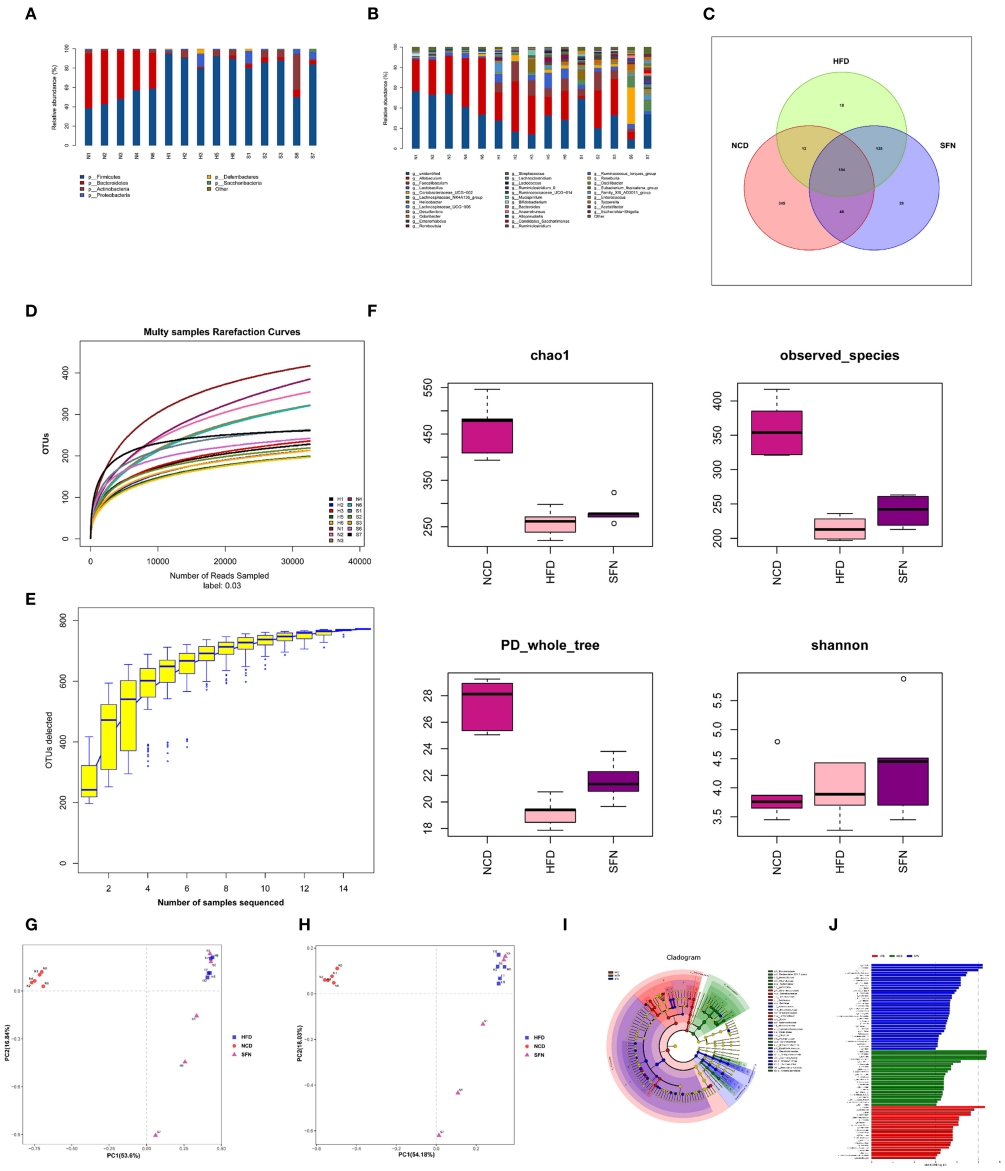
SFN intervention manipulated the gut microbiota composition in HFD-fed mices, five animals from each group were analyzed. **(A)** At phylum level. **(B)** At genus level. **(C)** Venn diagram representation of the number of OTUs at the genus level from the NCD, HFD, and HFD+SFN group. **(D)** The rarefaction curves, **(E)** Species accumulation curves, **(F)** Chao1, Observed_species, PD_whole_tree, Shannon. **(G)** PCA based on the relative abundance of OTUs. **(H)** PCoA score plot based on Unifrac and Bray–Curtis. **(I)** Taxonomic cladogram derived from LEfSe analysis. **(J)** The species with LDA scores higher than the set value 3.

To evaluate the changes in the gut microbiota after SFN treatment, we conducted the *16S* rRNA sequencing analysis. Compared to the NCD group, the relative abundance of *Deferribacteres, Proteobacteria, Firmicutes*, and *Actinobacteria* from HFD mice increased at the phylum level. Interestingly, *Tenericutes* increased in the HFD+SFN group. Conversely, the abundance of *Deferribacteres* was low, which was similar to the NCD group. Moreover, the *Firmicutes*-to-*Bacteroidetes* ratio was significantly higher in HFD-fed mice (84.182) compared to the NCD-fed mice (1.028), but the ratio was decreased to 16.189 by SFN treatment. At the genus level, HFD increased the abundance of *Mucispirillum, Helicobacte*r, *Lachnospiraceae_UCG_006, Lactobacillus*, and *Romboutsia* and decreased the levels of *Bifidobacterium, Bacteroides, Ruminococcaceae_UCG-014, Ruminococcaceae_UCG-010, Prevotellaceae_UCG-001*, and *Eubacterium_fissicatena_group*. However, SFN increased the relative abundance of *Bifidobacterium, Romboutsia*, and *Ruminococcaceae_UCG-014* (Figures 4A,B). The Venn diagrams indicated that the 184 operational taxonomic units (OTUs) were identical among the three groups (OTUs). The HFD group demonstrated the most unique genus (18 OTUs) followed by the NCD group (345 OTUs) and HFD+SFN group (29 OTUs) (Figure 4C). Together, these findings indicated that SFN regulates the composition of gut microbiota in HFD-fed mice.

As shown in Figure 4D, the rarefaction curve progressed to a plateau, which indicated that the gut microbial diversity in all the samples was captured at the current sequencing depth. The *Specaccum* species accumulation curves tended to flatten with the increase in sample size, indicating that the sampling was sufficient for data analysis (Figure 4E). For the microbial alpha diversity, the Chao 1, observed_species, Shannon and PD_whole_tree showed that SFN intervention increased the species diversity compared with the HFD group after administration of SFN for 6 weeks (Figure 4F).

Principal component analysis (PCA) and principal coordinate analysis (PCoA) based on Unifrac and Bray–Curtis distance were utilized to analyze the difference in the gut microbiota of each sample. As shown in Figures 4G,H, there was a large gap between the HFD group and NCD group, suggesting that HFD exerts a notable effect on the composition of gut microbiota. Although there was some overlap, a certain distance was also observed between the HFD and HFD+SFN groups, indicating specific differences in the composition of gut microbiota between the two groups. Furthermore, specific bacterial taxa with varied relative abundance were identified among NCD, HFD, and HFD + SFN groups by linear discriminant analysis (LDA) effect size (LEfSe) analysis (LDA threshold is 3). As shown in Figures 4I,J, the major species in the NCD group were *Bacteroidetes*, at the genus level, *Bacteroidetes* and *Alloprevotella*, and at the family level, *Prevodiaceae* and *Bacteroidales_S247_group*. The species that play a significant role in the HFD group are *Deferribacteres* and *Firmicutes*. At the genus level, *Facecalibaculum, Romboutsia*, Lachnospiraceae_UCG_006, and Streptococcus, and at the family level, *Peptostreptococcaceae, Streptococcaceae*, and *Deferribacteraceae* were abundant. The key species of the HFD+SFN group were *Clostridia* class and *Clostridiales* order.

### SFN Administration Altered the Level of IAA Metabolite in HFD-Fed Mice

**Figure 5:**
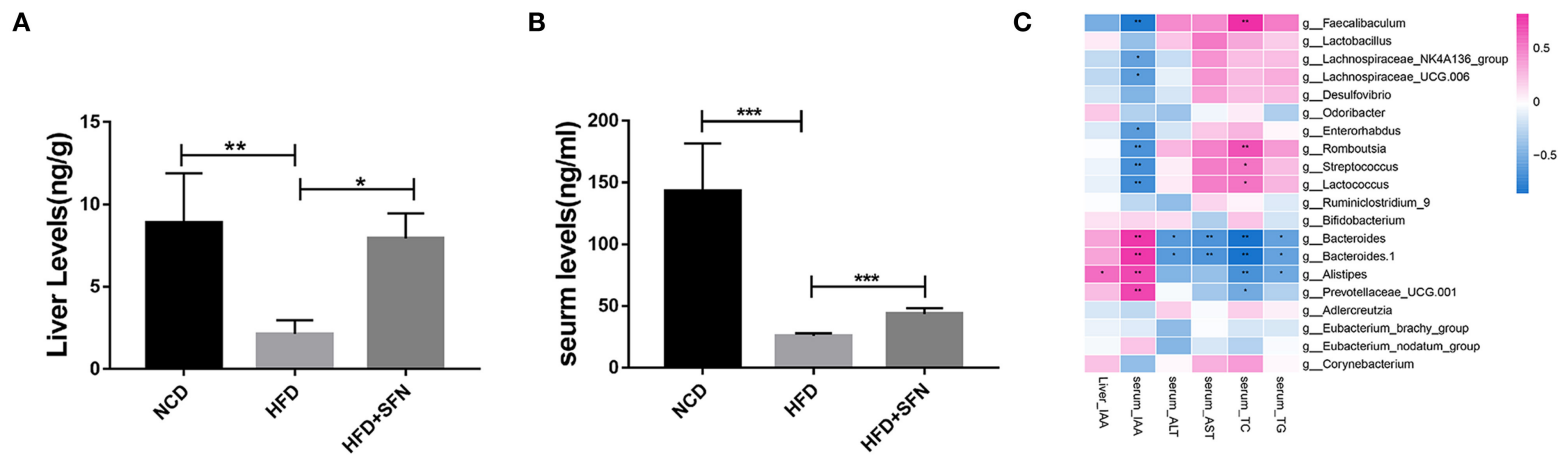
IAA concentrations in the liver **(A)** and serum **(B)** from mice of different groups. **(C)** Heatmap of the Spearman r correlations between the gut microbial species and biomarkers. Data are presented as mean ± SD. *P < 0.05, **P < 0.01, ***P < 0.001 vs. HFD group.

To assess the metabolic changes in the gut microbiota remodeled by SFN, we performed targeted UPLC-MS/MS experiments to detect the content of IAA in serum and the liver of mice in the NCD group, the HFD group, and the HFD+SFN group. These analyses indicated that the levels of IAA in the liver and serum of HFD mice were notably lower than that of NCD mice but increased significantly by SFN (*P* < 0.05, *P* < 0.0001) (Figures 5A,B).

To further explore the relationship between metabolite IAA and gut microbiota, as shown in the Sperman heatmap (Figure 5C), the abscissa is the differential metabolite, and the ordinate is the different species. Red represents positive correlation, blue represents negative correlation, and the darker the color, the stronger the correlation. *Bacteroides, Alistipes* and *Prevotellaceae_UCG.001* were positively correlated with serum IAA and negatively correlated with serum total cholesterol (TC). However, *Romboutsia, Streptococcus, Lactococcus* and *Faecalibaculum* were positively correlated with serum TC and negatively correlated with serum IAA.

### Sulforaphane Attenuates the Expression of Proinflammatory Cytokines in Macrophages

**Figure 6:**
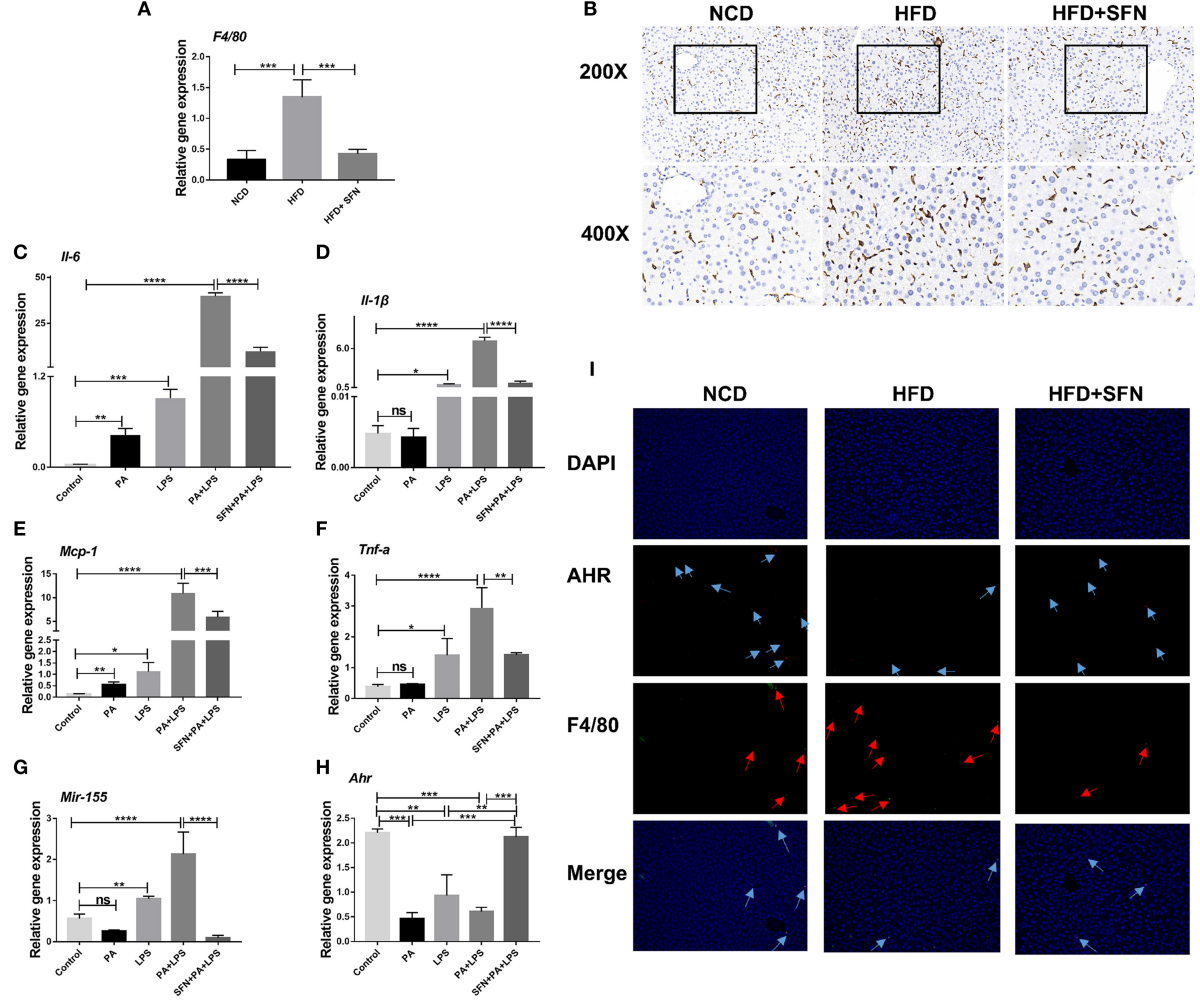
SFN inhibited the liver inflammation effects and attenuated the expression of proinflammatory cytokines in macrophages. **(A)** The mRNA expression of *F4/80* in the liver tissue. **(B)** IHC staining displayed the expression of F4/80 (brown particles), in the livers of different groups. **(C–F)** Changes in the expression of *Il-6, Il-1*β, *Mcp-1*and *Tnf-a*. **(G)** *Mir-155* expression in Raw264.7 cells. **(H)** mRNA expression of *Ahr*. **(I)** AHR RNA-ISH combined with F4/80 IF staining for macrophages of mouse liver sections. Arrows indicate AHR RNA and F4/80, respectively. Original magnification, 200×. Data are presented as mean ± SD. ns, not significant, *P < 0.05, **P < 0.01, ***P < 0.001, ****P < 0.0001 vs. the Control group or PA+LPS group or HFD group.

The mRNA expression of *F4/80* (macrophage surface marker) was inhibited by SFN in the liver of HFD mice (Figure 6A), and IHC showed similar results (Figure 6B). These results demonstrated that SFN reduced the number of macrophages in the liver and inhibited liver inflammation.

Furthermore, we treated Raw264.7 cells with PA and LPS to simulate the two key factors affecting the development of NAFLD to non-alcoholic steatohepatitis (NASH). Then, the expression of *Il-6, Il-1*β, *Mcp-1, Tnf-a*, and *Mir-155* increased significantly after PA, LPS, and PA+LPS-treatment, whereas these mRNA expression were suppressed by SFN (Figures 6C–G). Conversely, the mRNA expression of *Ahr* was clearly increased by SFN treatment (Figure 6H), while the results of AHR in RNA-ISH showed that the AHR expression in the macrophages of the liver was also upregulated under SFN administration. Similarly, RNA-ISH combined with F4/80 IF staining for macrophages of mouse liver sections showed that the expression of F4/80 decreased in the HFD+SFN group, which was consistent with the results of IHC (Figure 6I).

## Discussion

Gut microbiota plays pivotal roles in the onset and progression of NAFLD and exert a marked impact on host metabolism (30, 31). Gut microbial tryptophan metabolites have been confirmed as ligands of AHR and influence the progression of obesity, type 2 diabetes (T2D), hypertension, inflammatory bowel disease (IBD), multiple sclerosis (MS), and Huntington's disease (HD) (16). However, HFD alters the structure and metabolic ability of intestinal microflora in mice, which further decreases the level of gut microbiome-derived tryptophan metabolites (3, 32, 33). In addition, AHR contributes to the establishment of intestinal microbiota in mice, and the cecal microbiota of Ahr^−/+^ and Ahr^−/−^ mice show different macrogenomic metabolic pathways (34). In summary, the interaction among gut microbiota, tryptophan catabolites, and AHR may plays an essential role in the development of NAFLD. However, whether metabolite IAA levels are correlated with the efficacy of drug interventions is yet to be elucidated. Herein, we investigated the diet-related AHR agonists that improved NAFLD by regulating gut microbiome-derived metabolites.

**Figure 7:**
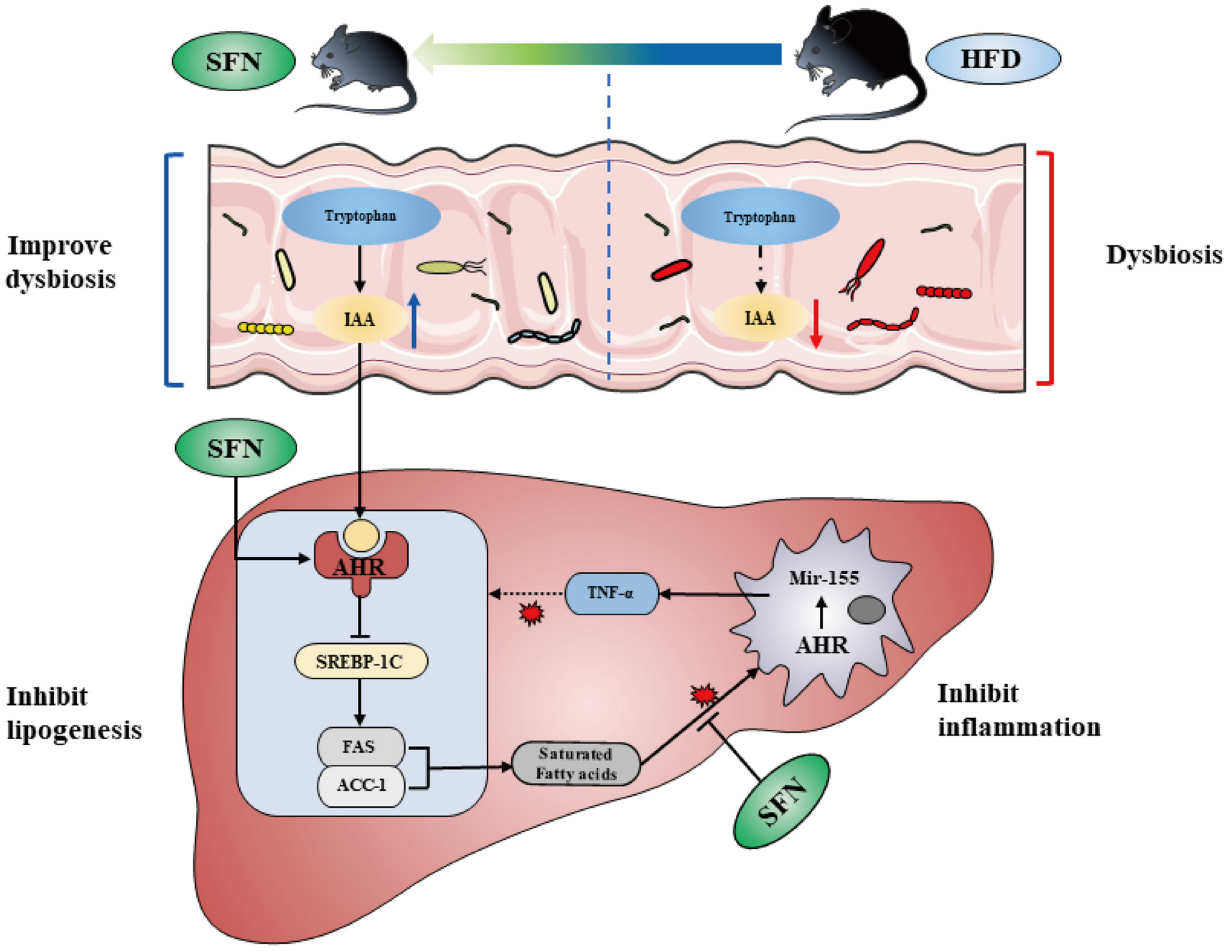
SFN altered the gut microbiota of mice and microbiome-derived metabolite IAA. Additionally, SFN regulated liver lipid metabolism by activating AHR and inhibited chronic low-grade inflammation.

In the present study, we find the therapeutic capacity and improvement activity of sulforaphane against NAFLD can be mediated by metabolic and immune pathways (Figure 7).

Previous studies reported that SFN regulates lipid metabolism (35). The current data demonstrated that SFN significantly decreased the levels of serum TC and TG. In addition, SFN inhibited the *de novo* lipogenesis in both mouse and HepG2 cell experiments. These results further confirmed that SFN improves lipid metabolism.

Reportedly, AHR is distributed in various tissues and cell types, such as macrophages, Treg cells, dendritic cells (DCs), and innate lymphoid cells (ILC). However, AHR exerts different effects when activated by various ligands in different cell types, which leads to differences in the data (18). In addition, the activation of AHR/CD36 pathway promotes hepatic steatosis in mice (36), while inhibiting the activity of AHR and altering the expression levels of CYP1B1, PPARα, and SCD-1 that attenuate the diet-induced obesity and hepatic steatosis in mice (37). On the contrary, the activation of AHR by IAA negatively regulates SREBP-1C and FAS, which in turn, improves NAFLD (3). These findings implied that AHR might have a dual role in regulating liver lipid metabolism. However, we demonstrated that SFN negatively modulates the expression of lipogenesis genes, *SREBP-1C, FAS*, and *ACC1* via AHR activation, which was consistent with the results of the IAA treatment group. As shown in previous studies, SFN activated NRF2, indicating tight bidirectional crosstalk between AHR and NRF2 (38, 39). These results suggested that SFN improves NAFLD by activating AHR to regulate lipid metabolism.

Targeted metabolomics profiles uncovered a prominent decrease in the levels of IAA in HFD mouse serum and liver tissues, which was consistent with previous results (3). Importantly, IAA not only delays NAFLD progression by activating AHR as an endogenous ligand but also alleviates it by reducing liver lipid production, oxidative stress, and inflammation (15). Nevertheless, SFN increased the level of IAA in this study. Accumulating evidence clarified that IAA is one of the foremost tryptophan metabolites produced by intestinal bacteria, such as *Clostridium, Bacteroides, Bifidobacterium, Eubacterium hallii, Peptostreptococcus, Asscharolyticus, Lactobacillus reuteri*, and *E. coli* (14, 40). Our data demonstrated that HFD decreased the abundance of *Bifidobacterium, Bacteroides, Eubacterium_fissicatena*_*group*, and *Erysipelatoclostridium*. However, the relative abundance of *Bifidobacterium* and *Bacteroides* was increased by SFN treatment. Additional studies have shown that *Gammaproteobacteria* and *Prevotella* are related to endogenous ethanol production (41). The intrahepatic *Proteobacteria* was associated with NASH, ballooning degeneration, lobular and portal inflammation, and liver fibrosis (42). Correlation analysis revealed that *Cyanobacteria* and *Bacteroides* were positively correlated with IL-10 and *Ferribacteres, Tenericutes, Mucispirillum*, and *Ruminiclostridi_6* were correlated with proinflammatory reactions (43). The present study showed that SFN adjusted the comparative abundance of the related microbiota described above. Accumulating evidence showed that the abundance of gut microbiota is elevated as a result of hepatic TG production (44). Therefore, SFN could not only adjust the structure and diversity of gut microbiota but also indirectly improve NAFLD by modulating the content of gut microbiome-derived metabolite IAA.

IL-1β produced by macrophages recruit the inflammatory factors to the liver and activate hepatic stellate cells, thereby developing liver fibrosis. IL-1β also promotes adipogenesis and TG accumulation in hepatocytes by regulating the expression of Srebp-1c, which together with TNF-α triggers hepatocyte necrosis (45–47). In this study, SFN treatment reduced the mRNA expression of proinflammatory cytokines as well as upregulated the mRNA expression of *Ahr* in Raw264.7 cells. Another recent study identified that AHR negatively regulates the expression of LPS-induced inflammatory cytokines (48). In addition, the mRNA expression level of *Ahr* in peritoneal macrophages increased at 1h post-LPS treatment, reached a peak at 2 h, and then declined gradually (49). Therefore, the mRNA expression of *Ahr* in macrophages decreased after PA+LPS stimulation for 24 h, and increased in the liver tissue of HFD mice, which might be related to time.

In conclusion, the current findings demonstrated that SFN altered the gut microbiota and microbiome-derived metabolite IAA of mice and regulated the liver lipid metabolism by activating AHR. These results may have important implications for unraveling the metabolic basis of the disease and provides evidence that modification of the intestinal microbiota could be therapeutic for the amelioration of hepatic steatosis in obese individuals.

## Data Availability Statement

The datasets presented in this study can be found in online repositories. The names of the repository/repositories and accession number(s) can be found at: https://www.ncbi.nlm.nih.gov/search/all/?term=PRJNA763001; Sequence Read Archive (SRA) database (SAMN21419598-SAMN21419612).

## Ethics Statement

The animal study was reviewed and approved by the Institutional Review Board (or Ethics Committee) of Radiation Medicine Chinese Academy of Medical Sciences.

## Author Contributions

XX, SS, and LL carried out the studies and drafted the manuscript. CL, QH, MR, LZha, and JingyueZ participated in collecting data. CY, HY, LZho, XC, and XD performed the statistical analysis and participated in its design. JieZ, JingwenZ, and BW helped to draft the manuscript. All authors read and approved the final manuscript.

## Funding

This work was supported by National Key R&D Program of China [Grant No. 2019YFC0119505]; National Natural Science Foundation of China [Grant No. 81970477]; Natural Science Foundation of Tianjin [Grant No. 18JCQNJC80700]; Tianjin Science and Technology Innovation Cooperation Project [Grant No. 19PTZWHZ00090]; and Tianjin Research Innovation Project for Postgraduate Students [Grant No. 2019YJSS188].

## Conflict of Interest

The authors declare that the research was conducted in the absence of any commercial or financial relationships that could be construed as a potential conflict of interest.

## Publisher's Note

All claims expressed in this article are solely those of the authors and do not necessarily represent those of their affiliated organizations, or those of the publisher, the editors and the reviewers. Any product that may be evaluated in this article, or claim that may be made by its manufacturer, is not guaranteed or endorsed by the publisher.

## Abbreviations

AHR: aryl hydrocarbon receptor
LPS: lipopolysaccharide
SFN: sulforaphane
IAA: indole-3-acetic acid
HFD: high-fat diet
AST: aspartate aminotransferase
ALT: alanine aminotransferase
SREBP-1c: synthase and sterol regulatory element-binding protein-1c
NRF2: NF-E2-related factor 2
FAS: fatty acid synthase
SCD1: stearoyl-CoA desaturase-1
NQO1: NAD(P)H: quinine oxidoreductase 1.
